# Oil Spills and Human Health: Contributions of the Gulf of Mexico Research Initiative

**DOI:** 10.1029/2019GH000217

**Published:** 2019-12-11

**Authors:** Ruth L. Eklund, Landon C. Knapp, Paul A. Sandifer, Rita C. Colwell

**Affiliations:** ^1^ Masters in Environmental and Sustainability Studies Program College of Charleston Charleston SC USA; ^2^ Center for Coastal Environmental and Human Health College of Charleston Charleston SC USA; ^3^ University of Maryland MD USA; ^4^ School of Public Health Johns Hopkins University Baltimore MD USA

**Keywords:** GoMRI, Gulf of Mexico Research Initiative), oil spill, human health, health research, Deepwater Horizon

## Abstract

The Gulf of Mexico Research Initiative (GoMRI) was established in 2010 with $500 million in funding provided by British Petroleum over a 10‐year period to support research on the impacts of the Deepwater Horizon oil spill and recovery. Contributions of the GoMRI program to date focused on human health are presented in more than 32 peer‐reviewed papers published between 2011 and May 2019. Primary findings from review of these papers are (i) the large quantity of dispersants used in the oil cleanup have been associated with human health concerns, including through obesogenicity, toxicity, and illnesses from aerosolization of the agents; (ii) oil contamination has been associated with potential for increases in harmful algal blooms and numbers of pathogenic *Vibrio* bacteria in oil‐impacted waters; and (iii) members of Gulf communities who are heavily reliant upon natural resources for their livelihoods were found to be vulnerable to high levels of life disruptions and institutional distrust. Positive correlations include a finding that a high level of community attachment was beneficial for recovery. Actions taken to improve disaster response and reduce stress‐associated health effects could lessen negative impacts of similar disasters in the future. Furthermore, GoMRI has supported annual conferences beginning in 2013 at which informative human health‐related presentations have been made. Based on this review, it is recommended that the Oil Pollution Act of 1990 be updated to include enhanced funding for oil spill impacts to human health.

## Key Points

- Nearly 3% of >1,200 Gulf of Mexico Research Initiative publications were directly related to human health, a higher proportion than previous oil spill research
- Dispersants contained possible obesogens and decreased aerosolized volatile organic compound exposure but increased particulate matter concentrations
- Those who rely on natural resources are more vulnerable to oil spills and cleanup methods that may increase exposure to harmful chemicals, algae, and bacteria

## 1 Introduction

On 20 April 2010, the largest oil spill in U.S. history to that time began when the Deepwater Horizon (DWH) drilling rig failed. This failure resulted in fires and subsequent explosion of the rig, taking the lives of 11 workers on board and releasing approximately 4.9 million barrels (~205 million gallons) of crude oil into the Gulf of Mexico (GOM) over a period of 3 months (Tao et al., 2011). The cleanup response was extensive, requiring thousands of workers, including professional fishermen, engineers, technicians, and scientists. Response efforts involved continuous submersible operation (MacDonald et al., 2014), the use of approximately 835 skimmers and 9,000 vessels, and declarations of states of emergency in each of the GOM States (US Coast Guard, 2011). Beaches were closed, oiled wildlife were collected, and extensive precautions were taken to prevent seafood contamination, including closure of commercial and recreational fisheries over ~37% of GOM federal fisheries waters (see Section 4; US Coast Guard, 2011).

Oil contamination is not only a concern for the environment but also for human health. However, effects of crude oil on human health have not been well studied (Woodward, 2010), although recent efforts such as the National Institutes of Health (NIH) GuLF STUDY (Kwok et al., 2017) and the Coast Guard Study (Rusiecki et al., 2018) are adding much new information. Within a few weeks after the DWH oil spill, British Petroleum (BP) announced a $500 million fund for research on the environmental and health impacts of the spill. The 10‐year‐long Gulf of Mexico Research Initiative (GoMRI) was created as an independent entity responsible to use those funds for research and education related to the spill (http://gulfresearchinitiative.org/). In its first years of operation, as its organizational structure was being established, block grant funding was awarded to each of the five Gulf States and to the National Institute of Environmental Health Sciences (NIEHS), totaling $45 million, to initiate research and establish baseline data for subsequent studies. This initial funding included money to support a Coast Guard cohort study (Rusiecki et al., 2018). The research supported by this $45‐million award was not under the overview of the GoMRI and so was not considered in this review. GoMRI issued its first call for research proposals in January 2011 and, employing a peer review process similar to that of the National Science Foundation, awarded $1.5 million in grants for data collection and monitoring. In the first of a series of formal requests for proposals (RFP‐I) in August 2011, funding totaling $110 million to be spent over 3 years was awarded to eight research consortia. The second request for proposals, RFP‐II, resulted in awards of $18.5 million in August 2012 for 19 research projects focused on GoMRI's five research themes. To fill a funding gap between 1 July 2011 and 30 September 2011, RFP‐III granted $1.5 million in bridge grants to 17 projects to ensure vital continuity of sampling. In January 2015, RFP‐IV awards totaled $140 million that was provided to 12 research consortia. In a shift to individual investigator proposals the following year, 22 proposals amounting to $38 million were funded in January 2016 under RFP‐V, with public health proposals receiving the largest proportion of the overall award. In January 2018, a total of $50 million in grants funded 31 research proposals (a mix of consortia and individual investigator projects) under RFP‐VI (GoMRI, n.d.). Additional information about GoMRI and a complete list of GoMRI‐supported publications can be accessed from its website (https://gulfresearchinitiative.org).

The five GoMRI research themes for which funds were provided were “physical distribution, dispersion, and dilution of petroleum (oil and gas), its constituents, and associated contaminants (e.g., dispersants) under the action of physical oceanographic processes, air sea interactions, and tropical storms; chemical evolution and biological degradation of the petroleum/dispersant systems and subsequent interaction with coastal, open‐ocean, and deep‐water ecosystems; environmental effects of the petroleum/dispersant system on the sea floor, water column, coastal waters, beach sediments, wetlands, marshes, and organisms, and the science of ecosystem recovery; technology developments for improved response, mitigation, detection, characterization, and remediation associated with oil spills and gas releases; and **impact of oil spills on public health, including behavioral, socioeconomic, environmental risk assessment, community capacity and other population health considerations and issues**” (GoMRI, n.d.; emphasis added).

As GoMRI neared the end of its 10‐year lifespan, the Research Board funded a series of workshops and other activities to review scientific progress made through GoMRI's efforts since the DWH spill in 2010 and commissioned synthesis of eight core areas of research, including human health.

GoMRI employed “public health” in its original statement of research themes and as one of the organizing headings for publications, but “human health” was subsequently used to describe one of the synthesis core areas. Human health is a more encompassing term, including public (population) health, and health and well‐being of individuals, families, and groups and encompasses both the psychological and physiological health of those different units of study as well as the interrelationships occurring among them. We, therefore, chose to use human health to describe the focus of our study.

Our literature review provides a summary of human health‐oriented publications resulting from research funded by GoMRI and specifically identified as public health contributions on the GoMRI website (Supporting Information Table S1). Papers not identified by GoMRI as resulting from its funding and having a human health focus were not included. In addition, GoMRI was the principal supporter of the Gulf of Mexico Oil Spill and Ecosystem Science (GOMOSES) conferences initiated in 2013 and held annually thereafter. The GOMOSES forum provided an additional method for GoMRI to support human health research outside of direct funding, through the dissemination of findings, and is therefore included in this synthesis. The number of oral and poster presentations on human health at the GOMOSES conferences were tallied as another metric of human health‐related contributions. Thus, the purpose of this paper is to summarize the contributions of GoMRI to human health research via the lens of health‐related publications that resulted from GoMRI funding and the numbers of health presentations that were enabled by GoMRI through the GOMOSES annual conferences it supported.

## 2 GoMRI Publications on Human Health

**Figure 1:**
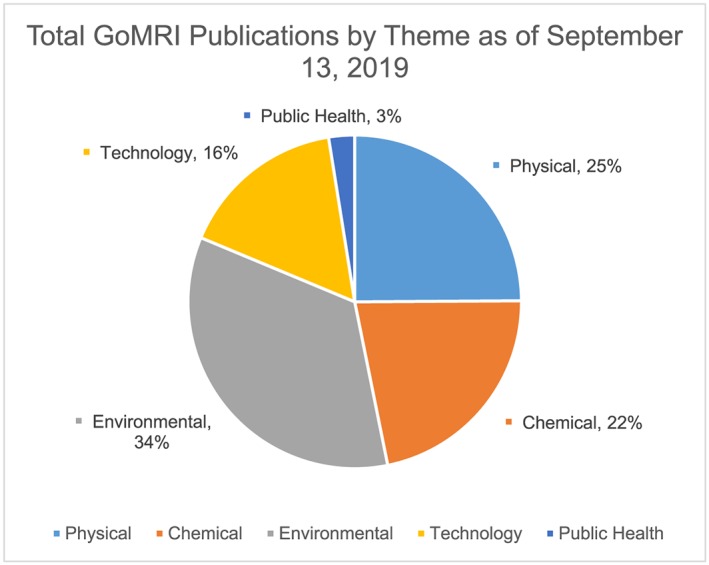
Total number of GoMRI‐funded publications within each research theme denoted by theme and percent of the total number of publications.

This review is restricted to peer‐reviewed publications that were listed on the GoMRI website under the Public Health heading. A total of 40 publications were identified as health‐themed research (http://research.gulfresearchinitiative.org/gomri-publications/, last accessed 01 May 2019; Table S1). A few additional papers have appeared or are in press for a total of 44 to date. Upon examination, 32 of these publications were found to be directly relevant to human health. As of 13 September 2019, GoMRI has supported 1,747 published or in‐press publications. Of these publications, the Public Health heading comprised only 3% of the total number of GoMRI‐supported research papers to date (Figure 1), but this small body of work contributes significantly to our understanding of how oil spills and remediation efforts impact public and human health. Our judgment that the papers supported by GoMRI, taken together, comprise a significant contribution to oil spill health research is based on our familiarity with a much broader literature on the topic, the fact that several different facets of health were included among the papers, and the relative high proportion of health‐focused papers compared to literature from previous oil spills (Murphy et al., 2016). Further, additional health‐related papers are in preparation or under review and not yet available for inclusion. For example, two currently funded projects are examining human lung models to evaluate effects of dispersants. Also, publications related to toxic effects of oil and dispersants on nonhuman organisms were not included but some may have indirect relevance to human health. Because these were beyond the scope of this review, our findings should be considered only a portion of the GoMRI contribution to the human health literature. In addition, the funds included in the first year of block funding for the NIEHS were not under GoMRI Research Board oversight. Hence, any publications resulting from that support were not included in this review.

For purposes of this review, the literature is grouped into four general categories: dispersants, oil contamination, human studies, and disaster planning and response. Studies with overlapping categories are grouped according to their primary focus. We employed this organizational scheme to improve the flow of the paper and make it easier for readers to find information of particular interest to them.

## 3 Dispersants

The marine ecosystem of the GOM absorbed a “very large dose of toxic hydrocarbons and dispersant” as a result of the DWH oil catastrophe (MacDonald et al., 2014, p. 1). Cleanup efforts utilized a variety of methods including surface and subsurface applications of two chemical dispersants, Corexit 9500 and 9527A, to break up the oil. More than 1.8 million gallons of Corexit were released throughout the GOM, prompting scientific inquiry regarding effects of dispersant‐oil mixtures on human and marine populations. Human‐related studies focused on potential impacts of aerosolized oil‐dispersant mixtures and the possibility that dispersants had obesogenic (promoting obesity in humans) effects.

Dispersants used on large oil spills reduce interfacial tension between seawater and oil, leading to increased marine aerosol emissions. Afshar‐Mohajer et al. (2018) studied aerosolization of volatile organic compounds (VOCs) and magnification of marine aerosol emissions after an oil spill. Researchers used a 6 × 0.3 × 0.6‐m tank to simulate wave patterns colliding with oil slicks. To determine aerosol particle size distribution, tests were conducted measuring seawater with crude oil slicks, crude oil and dispersant mixtures, and dispersant only (Afshar‐Mohajer et al., 2018). Results revealed that seawater mixed with crude oil only showed similar distribution of submicron particle sizes to that of seawater free of contaminants, but addition of dispersants mixed with crude oil to seawater decreased VOC concentration by two to three times while increasing the concentration of nanoparticles across all nano sizes measured (Afshar‐Mohajer et al., 2018). The authors noted that reduction in water surface tension is responsible for the increased concentration of aerosolized particles, explaining why dispersant‐oil mixtures are found to have highest concentration of particulate matter (PM). Their results suggest that the human health concern of aerosolized VOCs is reduced following dispersant application but point to other possible human health risks related to PM inhalation and subsequent buildup in the pulmonary system when dispersants are used in remediation efforts.

In a following study, Afshar‐Mohajer et al. (2019) did a health risk assessment of aerosolized VOCs and fine PM inhalation. Adding dispersant (1:25) to crude oil slicks on water surfaces resulted in lower VOC concentration and “dispersant helped reduce the cancer risk of exposure to benzene, the main carcinogen emitted from the crude oil slick” (Afshar‐Mohajer et al., 2019, p. 931). While the lifetime cancer risk was reduced from 57 to 37 cases per million when using dispersants, potential exposures were still above the National Institute for Occupational Safety and Health acceptable risk limit. On the other hand, addition of dispersant increased PM inhalation, magnifying the human respiratory system burden by a factor of 10. Findings indicate that the use of dispersants confer disparate influences to human health risks, with their addition to crude oil resulting in reduction of health risks from VOCs while increasing the respiratory burden from PM inhalation. However, the health threats from VOCs remained much higher than those posed by PM inhalation, both before and after application of dispersants. The authors noted that times of exposure over 1 hr led to exceedances in several health thresholds and thus recommended caution should be taken by those in close proximity to seawater contaminated with oil.

Baatz et al. (2014) advanced methodology for analyzing respiratory impacts by developing a new and innovative method for rapid cryopreservation of human lung tissue, using a process of “pseudo‐diaphragmatic expansion of pieces of fresh lung tissue with cryoprotectant formulation,” abbreviated as PDX‐CP, followed by cryostorage (Baatz et al., 2014, p. 411). This new method can enhance biospecimen banking of lung tissue, making more materials available for research, particularly in relation to lung cancers. Although not specifically mentioned by the authors, their method may also be useful in providing material for future studies of pulmonary impact of inhaled oil components and oil‐dispersant mixtures on humans exposed to an oil spill.

Traditional models of decreasing caloric intake while increasing physical activity have dominated obesity research (Grün & Blumberg, 2006). However, mounting evidence of environmental exposures linked to obesity has inspired research into potential obesogens in the environment. Under the environmental obesity model, Grün and Blumberg (2006, p. S50) define obesogens as “molecules that inappropriately regulate lipid metabolism and adipogenesis to promote obesity.” Oil and dispersants have been reported to be potential endocrine disruptors (Adedara et al., 2014), and Ramachandran et al. (2004) found that dispersants increased the bioavailability of oil components, such as polycyclic aromatic hydrocarbons (PAHs), in fish. Water samples were collected on two separate occasions, by the team on the NOAA research vessel *Ocean Veritas* (27–29 May and 2–4 June 2010), from the surface and at various depths in the GOM (Gray et al., 2014). Corexit dispersants were identified and calculated to be in the 100–200 ppb range in the water column (Gray et al., 2014; Kujawinski et al., 2011), strongly suggesting a need for research into potential exposure effects on humans.

Bowers et al. (2016) and Temkin et al. (2016) explored the possibility that dispersants used in the DWH cleanup effort may act as an obesogen for humans. Both investigator teams focused on a probable obesogen, dioctyl sodium sulfosuccinate (DOSS), a component of Corexit, the main dispersant mixture used in DWH cleanup (Rusiecki et al., 2018). Because the nuclear receptor, peroxisome proliferator‐activated receptor gamma (PPARγ), is vital to adipocyte differentiation and is the main target of obesogens, Temkin et al. (2016) used PPARγ transactivation assays to identify putative obesogens. In laboratory experiments employing mice, DOSS was found to activate the PPARγ gene, leading to the conclusion that DOSS is a potential obesogen (Temkin et al., 2016).

Two components of Corexit, Span 80 and DOSS, are surfactants also commonly used in food and drink products (U.S. Food and Drug Administration, n.d.), air fresheners, and personal care products (U.S. Department of Health and Human Services, 2018). Bowers et al. (2016) tested DOSS and Span 80 for additive effects promoting adipogenesis and thereby, obesity. Experiments were conducted using cell cultures in the laboratory. DOSS and Span 80 were found to work synergistically, resulting in substantially “more adipocyte differentiation than treatment with either compound individually” (Bowers et al., 2016, p. 65). These results highlight the potential of unintended adverse side effects when DOSS and Span 80 are used as food additives and in dispersants to treat oil spills and point to the need to understand possible human health effects, if any, from oil spills treated with dispersants.

## 4 Oil Contamination

Many environmental contamination studies were conducted following the DWH oil spill in the GOM. DWH spill‐driven contaminants have been linked to pulmonary health issues in human populations (Lenes et al., 2013; Walsh et al., 2016) and shown to be a cause of fish kills (Dickey & Huettel, 2016) and contamination of seafood (Smith et al., 2011; Tao et al., 2011; Xia et al., 2012). GoMRI‐funded studies have also focused on oil spill impacts linked to the Florida red tide organism, *Karenia brevis*, pathogenic *Vibrio* bacteria, and seafood safety (Dickey & Huettel, 2016; Lenes et al., 2013; Smith et al., 2011; Walsh et al., 2017). Harmful algal blooms (HABs) in the form of *K*. *brevis*, and possible contamination with *Vibrio* spp., are considered health risks for human populations in the GOM.

HABs occur when algal populations proliferate, resulting in detrimental effects on humans and marine life. Spill‐driven contaminants can cause trophic cascades, contributing to these blooms. Weisberg et al. (2016) studied HAB formation by *K*. *brevis* on the west coast of Florida where the algal bloom occurs regularly between Tampa and Charlotte Harbor. They were able to confirm that *K*. *brevis* blooms originate offshore and move to near shore waters via subsurface onshore advection. Weisberg et al. (Weisberg et al., 2016, p. 118) found that upwelling was a “necessary condition for bloom delivery near shore” but that strong upwelling could result in diminution of a bloom since it allows for faster growing diatoms and other phytoplankton to outcompete *K*. *brevis*. They also noted that eutrophication (excessive nutrient pollution) of near shore waters likely contributes to bloom intensification but is not the primary initiating factor.

Release of toxic compounds (brevetoxins) by *K*. *brevis* during a red tide bloom causes fish kills, harms marine mammals and other species, and is highly dangerous for humans as well. Water turbulence and biological processes result in lysis of *K*. *brevis* cells and the subsequent release of brevetoxins into the water column. Following cell lysis, toxins accumulate in the water, aerosolize, and are transported by winds, causing or exacerbating asthma attacks and chronic obstructive pulmonary disease among those living in coastal regions (Lenes et al., 2013). In laboratory studies, Lenes et al. (2013) found that rats inhaling brevetoxins produced by *K*. *brevis* (PbTx‐3) were left with reduced antibody formation and an impaired immune system. They noted that asthmatic humans with PbTx‐3 immunosuppression were found to be subject to fatal pneumonia. Walsh et al. (2016, p. 69) termed these landward‐dispersed aerosolized toxins “marine HAB triggers” for asthma and chronic obstructive pulmonary disease. They estimated that ~48% of carbon sequestered in HAB cells on the West Florida Shelf became aerosolized and transported in wind‐blown spray.

Walsh et al. (2017) conducted a global analysis of how reduced zooplankton populations and consequently lowered phytoplankton grazing rates, accompanied by a variety of anthropogenic impacts, including overfishing and oil spills, resulted in increases in HAB formation and aerosolization of HAB toxins. They estimated that landward dispersion of aerosolized HAB toxins was responsible for 15% of asthma events worldwide in 2004, affecting ~45 million people and perhaps killing as many as 33,000. In addition, they identified the potential for HAB aerosols to also carry methyl mercury (MeHg) and other contaminants and suggested that as much as 40% of mercury poisonings from marine sources may be derived from HAB aerosols rather than from fish consumption. These estimates suggest much more significant and broad scale health threats from HABs than previously recognized. Walsh et al. (2016) concluded that plankton levels are out of balance within the GOM due to overfishing, release of heavy metals and pesticides, and oil spills, with likely results being a trophic cascade and a drastic increase in pulmonary issues for those nearby. Not surprisingly, they also pointed to the need for operational HAB forecasting to aid in the prevention of pulmonary diseases (Walsh et al., 2016).

The DWH oil spill caused an increased concern about shellfish contamination in the GOM (Dickey & Huettel, 2016). *Vibrio* spp. are relatively well‐studied bacteria because they include important human pathogens, occur naturally in coastal waters, play critical roles in the carbon cycle, and are highly efficient decomposers, including of some components of oil (Tao et al., 2011). *Vibrio parahaemolyticus* causes gastrointestinal illnesses and is the leading cause of seafood‐borne illness from consuming raw, improperly cooked, or contaminated seafood (Smith et al., 2011). Concern that the oil spill might encourage blooms of *V*. *parahaemolyticus* and lead to an increased risk of shellfish contamination post spill prompted Smith et al. (2011) to assess the response of *V*. *parahaemolyticus* strains local to the GOM to PAHs of crude oil. They assessed growth responses of 17 *V*. *parahaemolyticus* isolates from coastal Louisiana on two PAHs and found that neither encouraged growth of the bacteria and that a degradation product of one of the PAHs inhibited *V*. *parahaemolyticus* growth. Their work confirmed previous research that had suggested little additional risk from *V*. *parahaemolyticus* related to oil spills.

In a related study, Tao et al. (2011) examined weathered oil that washed up on Mississippi and Alabama beaches (forming tar balls) to determine if the highly pathogenic bacterium *Vibrio vulnificus* was associated with tar balls. *V*. *vulnificus* is of particular concern because it is the primary cause of seafood‐based human mortality in the United States. Contact with *V*. *vulnificus* in seawater can infect wounds and abrasions, with fatality rates of 20–30% (Tao et al., 2011). These researchers found not only that counts of total aerobic bacteria were “significantly higher in tar ball samples than in any other sample analyzed” (Tao et al., 2011, p. 509) but that levels of *V*. *vulnificus* in tar balls were 10 times higher than observed in sand samples and up to 100 times higher than in seawater. This information suggests a potentially severe health threat to humans who may come in contact with tar balls, as direct contact of *V*. *vulnificus* with even small open wounds can lead to life‐threatening infections (Tao et al., 2011). This finding may serve as the first identification of tar balls as a potentially serious human health risk for infectious disease.

Huettel et al. (2018) studied degradation of buried oil post‐DWH spill on Florida's Pensacola Beach and the role of tidal pumping in oil degradation. They noted the importance of maintaining oxygenated environments for microbial degradation of oil on beaches, citing previous reports that microbial degradation of oil occurs quickly in aerobic environments but nearly stops under anaerobic situations, such as in submerged microbial mats. Huettel et al. found abundance of bacteria in oiled sand increased by a factor of two, while bacterial diversity declined by ~50%, reflecting a bloom of oil‐degrading bacterial species. Importantly, they found that tidal pumping was a significant factor in oxygenating beach sediments, with a strongly positive effect on cleaning up oil deposited on Pensacola Beach. However, Huettel et al. also noted that oil buried on shore can be a source of PAHs for decades after a spill and that, even to the present day, residual surface tar balls wash ashore following storms and may result in human exposure to *V*. *vulnificus* bacteria (Tao et al., 2011) as well as probable obesogens (Temkin et al., 2016).

Several studies analyzed seafood contamination linked to the DWH spill. At the peak of the oil spill in June 2010, 88,552 square miles (37%) of Federal waters in the GOM were closed to fishing (US Coast Guard, 2011). The Federal closures were related to concern for potential contamination of seafood with oil components and depending on the area, fisheries were closed for 3–7 months, with a small area closed for a year. Xia et al. (2012) analyzed 278 seafood samples consisting of fishes, shrimps, crabs, and oysters collected weekly from 27 May 2010 until October 2010 and monthly until August 2011. These researchers compared PAH levels detected to the National Oceanic and Atmospheric Administration (NOAA) Mussel Watch program for the prior 10 years and found no significant difference in concentration. The PAH levels in GOM seafood were similar to levels found in grocery store and restaurant‐grade seafood and far below public health levels of concern established jointly by NOAA, the U.S. Food and Drug Administration, and Gulf Coast states (Xia et al., 2012). In a review paper that summarized seafood safety information obtained from 10,000 Gulf samples, including the work of Xia et al. as well as beach data, Dickey and Huettel (2016) reported that concentrations of potentially harmful oil components were at prespill levels soon after the end of the spill, although residual oil in waters and sediment could provide potential for ongoing exposures. They also noted concerns that existing seafood safety limits for oil compounds may not provide sufficient protection for certain vulnerable populations such as pregnant women, children, and ethnic groups that are high consumers of seafood (e.g., Vietnamese‐American fishers). Dickey and Huettel (p. 201) concluded that “(t)he DWH accident revealed the lack of adequate demographic and human health baseline data, benchmark environmental contaminant data, effective risk communication strategies, and accessible integrated surveillance systems linking human and environmental health status and trends.”

## 5 Psychosocial Studies

The DWH spill has been a long‐lasting life event for residents of the GOM, disrupting routine behavior, increasing anxiety, straining economic resources, and negatively affecting mental health (Ayer et al., 2019). Those employed in, and dependent upon, the oil and fishing industries were particularly vulnerable, suffering disproportionately during and after the disaster (Ayer et al., 2019; Cope et al., 2013; Cope et al., 2016; Cope & Slack, 2017; Lee & Blanchard, 2012; Parks et al., 2017).

Mental and behavioral health were studied in Gulf residents affected by the spill by Lee and Blanchard (2012), Cope et al. (2013), and Cope and Slack (2017), with emphasis on how community attachment affects mental health. Populations most vulnerable were those with high levels of economic, social, and cultural attachment to threatened, damaged, or depleted resources (Cope et al., 2013). According to the ecological symbolic theory, natural resource‐dependent communities experience long‐term stress impacts when renewable resources are endangered (Lee & Blanchard, 2012). Rural communities traditionally reliant on resource‐based economies have been shown to be particularly vulnerable to risks and disruptions associated with a disaster (Cope & Slack, 2017). Cope and Slack used county‐level demographic data and socioeconomic status to calculate an index of place‐based social vulnerability developed by Cutter et al. (2003) called the Social Vulnerability Index. Social vulnerability is asserted to be just as important as physical exposure to oil spills (Cope & Slack, 2017) in terms of effects on health and well‐being. Vulnerable populations were found to have increased psychological stress after the DWH spill (Cope et al., 2013; Lee & Blanchard, 2012), but researchers found that strong community attachment was connected to better outcomes for families, increased individual well‐being, and positive mental and physical health outcomes (Cope et al., 2013), but this may not hold for all situations.

Ayer et al. (2019) hypothesized that exposure to DWH would be related to depression, anxiety, alcohol abuse, and illness anxiety (defined by Ayer et al., 2019, p. 2) as “excessive concern or worry about having or getting a serious illness”) 6 years post spill. Researchers in that study analyzed impacts of other past traumatic experiences along with self‐reported exposure to the DWH spill to assess the influence of each factor on occurrence of mental health problems, weighting for gender and age. From 22 April to 6 August 2016, Ayer et al. conducted 2,520 telephone interviews in 45 coastal GOM counties. They found that trauma history was the most significant predictor for each of the outcomes. Yet, exposure to the DWH spill did not predict depression, anxiety, or alcohol use beyond past exposure to traumatic events but was significantly associated with illness anxiety.

Drakeford et al. (2019) explored religious influence on alcohol consumption in the wake of natural disasters such as the DWH spill. Environments with high levels of religious practice have long been thought to help mitigate disaster impacts for members through increased social support and enhanced social resources (Drakeford et al., 2019). However, recent research suggests highly religious contexts may have a negative impact on nonreligious community members, as they may experience difficulty gaining the same levels of support as religious members (Stroope & Baker, 2018). Drakeford et al. theorized such contexts may lead to increased maladaptive coping methods such as problematic alcohol consumption. To test this hypothesis, the authors utilized various data sources such as the Survey of Trauma, Resilience, and Opportunity in Neighborhoods in the Gulf (STRONG), the 2010 Religious Congregations Membership Study, 2012–2016 county‐level data from the American Community Survey 5‐year estimates, and 2016 County Business Patterns series data along with the Alcohol Use Disorders Identification Test and social inconvenience (defined as the interruption of individual behaviors that make up one's social life) measured through yes/no questions. The STRONG survey total sample included 2,520 adult residents of the GOM coastline, and the authors used weighted adjustments to adequately represent GOM resident demographics. After weighting, the mean Alcohol Use Disorders Identification Test score for the entire sample was 2.1, indicating that much of the sample was at risk for alcohol misuse with little difference between majority (2.2) versus minority (2.0) religious counties. However, highly religious counties reported higher levels of social inconveniences than their less religious county counterparts. Those who were less religious but living within highly religious communities reported higher levels of alcohol misuse along with increased DWH spill‐related social inconvenience (Drakeford et al., 2019). Findings indicate that while religious contexts are not overall associated with alcohol misuse, in some circumstances, highly religious contexts may actually increase alcohol misuse among nonreligious community members (Drakeford et al., 2019).

Parks et al. (2017, p. 278) analyzed “lifeway disruption” to determine the degree to which the DWH spill affected routine behaviors of people in impacted communities. According to the conservation of resources theory, those directly tied to the renewable resource‐based industries affected are more likely to have negative impacts. “The COR model suggests that chronic loss of or threat to such resources, especially when coupled with an inability to develop new or alternative resources, may result in a ‘loss cycle' for residents who are unable to adapt” (Parks et al., 2017, p. 279). Disruptions of routine behaviors were studied through analysis of results from the Louisiana Community Oil Spill Survey. The authors found that “respondents reported difficulty completing approximately one‐third of the activities” after the spill but those disruptions decreased over time indicating recovery (Parks et al., 2017, p. 283). Unsurprisingly, Parks et al. found that residents with a fishing/oil career background were shown to have greater life disruptions than their counterparts whose employment and way of life were not directly affected.

In an effort to evaluate how oil spill‐related resource loss is connected to mental and behavioral health issues, Ramchand et al. (2019) used STRONG survey data to compare Gulf states prevalence estimates of depression, anxiety, alcohol misuse screenings, and resident's health care usage 6 years post spill. Overall, nearly half of adults in this study screened positive for depression, anxiety, or alcohol misuse, while less than 20% of those individuals were engaging in mental health care. Among those screened positively for depression, anxiety, or alcohol misuse, women were more likely than men and lesbian, gay, or bisexual residents were more likely than nonlesbian, gay, or bisexual residents to have visited a mental health provider in the past 12 months. Those working in the fishing and seafood industry during the DWH spill were more likely to screen positive for depression, and higher levels of resource loss were positively correlated with depression and anxiety symptoms (Ramchand et al., 2019).

Disruptions in routine behavior following a disaster perpetuate long‐term consequences and increase stress surrounding the event. These social disruptions pose a threat to community attachment and could make recovering from a disaster in a timely manner more difficult. How well individuals and communities adapt to and recover from disaster‐related adverse conditions is measured in terms of resilience (Patel et al., 2018). In human health and disaster contexts, resilience can be understood as the relationship between risk and protective factors (Patel et al., 2018). Risk factors can include low socioeconomic status, poor education, past trauma, being of a minority group, and lack of an adequate support structure in addition to others, whereas protective elements include having a positive outlook, social ties, community involvement, spirituality, and others (Bonanno, 2004; Neenan, 2009).

Federal data indicate that approximately one in four shrimpers in the GOM are members of the Southeast Asian community (predominantly Vietnamese Americans; Macchi, 2015), a potentially vulnerable population after the DWH spill due to their natural resource dependence. Patel et al. (2018), however, hypothesized that Vietnamese Americans along the Gulf Coast would have higher levels of resilience because of their close community ties. Researchers examined the resilience levels of those identifying as Vietnamese within the Gulf Coast communities of Port Sulphur, LA, Galliano, LA, and Bayou La Batre, AL, through in‐person interviews and surveys using the 10‐item Connor Davidson Resilience Scale. Nearly the entire sample of those who identified as Vietnamese (98.67%, 74 out of 75 such participants) were from Bayou La Batre, AL, and those identifying as Vietnamese made up 24.2% of the total sample of 326 people (Patel et al., 2018). Results indicated that higher age and higher level of college education were associated with increased resilience, while identifying as Vietnamese was related to a 20% lower resilience score compared to other ethnic or racial identities. However, the authors did not control for natural resource dependency. Patel et al. concluded that further work is needed to understand the causes of apparent reduced resilience in the Vietnamese American population and to better support and equip such communities to deal with negative events.

Cope et al. (2016) studied how the DWH spill affected residents' blame and trust in institutions over a 3‐year period following the event. At each yearly anniversary of the rig explosion, the authors measured blame for the consequences of the disaster and distrust in three institutional actors—BP, federal government, and state government. Baseline data collected in October 2010 showed distrust in both BP and the federal government was similarly high among respondents in the study, while distrust in state government was significantly lower. Perceptions of blame at the baseline revealed that BP was firmly considered the primary responsible party (>75% of respondents), with the federal government serving as the second leading party for blame (~50%), and the state government much lower (~20%). Over the years of the study, there was no evidence that the views of BP or the federal government were trending better or worse over time for either trust or blame. However, perceptions of blame for state government increased from their low baseline level to ~32% of respondents by the study's end (trust remained stable). The authors additionally studied the effect of employment in natural resource occupations on perceptions of blame and trust, further dividing employment into renewable (fishing seafood) versus nonrenewable (oil gas). Taken cumulatively, those in natural resource occupations were more likely to blame BP and the federal government than those in other occupations. Households dependent on oil gas were more distrustful of the federal government than those in other industries with no significant difference in their trust of BP or state government. The authors attributed this finding to the combative relationship between the federal government and the oil‐gas industry, with a moratorium placed on extraction in the GOM and stricter regulations on the industry following the lift of that moratorium. Households dependent on the fishing‐seafood industry were more distrustful of all three institutional actors. While there was a perceived certainty of the reopening of the oil‐gas industry, the authors posited the unknown fate of the fishing‐seafood industry by the close of the study window (April 2013) was likely the cause of that distrust (Cope et al., 2016).

Petrun Sayers et al. (2019) used data from the STRONG telephone survey to measure the primary ways in which individuals obtained their news and their trust levels in these sources of information outside of a disaster context. Residents were most likely to obtain their news from television (41.53%) and the Internet (32.84%), with younger adults being more likely to report the Internet as their primary source of information. Minority populations were more likely to say that “it is very important to see their communities and people like them reporting the news,” and those with higher levels of education were more likely to place high trust in information from academic institutions and question news distributed through social media (Petrun Sayers et al., 2019, p. 11). Following national trends, younger adults (18–49) surveyed were found to be more critical of news passed through social media despite being heavily reliant on Internet channels. Young adults without higher education were 2.38 times more likely to report word of mouth as their primary communication method. Older adults aged 65+ were less likely to report the internet, radio, and word of mouth, preferring more traditional news sources such as television (Petrun Sayers et al., 2019).

## 6 Disaster Planning and Response

While the United States arguably has the best disaster planning and response systems in the world, these still have major limitations. Disasters recurring in the GOM pose complex scenarios for response agencies attempting to minimize negative impacts. Petrun Sayers et al. (2019) maintain that planning for these events should incorporate how and when to engage and include vulnerable populations.

Nicholls et al. (2017) pointed to the need for emergency management officials to recognize the ability and usefulness of community health workers (CHWs) in disaster planning and response. CHWs are already a part of the community, know the ins and outs, and are highly trusted. CHWs are able to contribute to public health, community resilience, and disaster preparedness and recovery through their unique position of living within the community in which they work. Nicholls et al. further recommended the use of a specialized public health and community training curriculum for CHWs developed and implemented by the University of Southern Alabama.

Sandifer and Walker (2018) reviewed disaster typology, recent disaster history, the resilience concept in relation to disasters, health impacts of disasters (especially those associated with chronic stress), and a variety of problems related to disaster response in the United States. Based on an extensive, targeted literature review, they made eight recommendations designed to enhance the resilience of both individuals and communities to disasters, including both long‐term and short‐term health effects: (1) improve disaster‐focused health programs and responses; (2) increase collection of predisaster health data, including biomarkers; (3) enhance capacity of science and health responders; (4) use natural infrastructure to reduce disaster impacts; (5) better include displaced persons in disaster responses; (6) utilize nature‐based treatment for disaster‐associated stress; (7) strengthen health focus in disaster‐related laws, policies, and regulations; and (8) develop more equitable process for dealing with financial aspects of disaster recovery to reduce recreancy.

Outreach programs have been focusing on broader impacts of scientific work in disaster response. In an effort to further science outreach, Beresford et al. (2018) shared recommendations for the development, management, and implementation of outreach plans. These recommendations for a science outreach program based upon GoMRI perspectives are (1) to ensure feasibility and efficacy, involve a multidisciplinary team at or prior to the proposal stage; (2) define the strategic plan and include well‐defined and measurable goals; (3) clearly define audiences and determine the most effective ways to reach them; (4) be familiar with team member skills and backgrounds and tailor outreach based on strengths of the team; (5) ensure that budgets and outreach plans are collaborative; (6) determine evaluation metrics at the beginning of the program; and (7) foster effective communication within the team and when communicating with constituents and other entities (Beresford et al., 2018).

Finucane et al. ([Link], p. 2) propose a self‐evaluative and adaptive systems approach to community resilience, asserting that “diverse perspectives on resilience can result in conflicting priorities before, during, and after disasters.” The Consortium for Resilient Gulf Communities (CRGC) uses an adaptive systems approach within the GOM based on building trust through exploring various knowledge types. Functions of the CRGC approach are (1) support stakeholders; (2) respond to stakeholder needs; (3) generate practical information, tools, and ideas for stakeholders to address specific problems and identify priorities; and (4) offer evidence‐based guidance to stakeholders (Finucane et al., [Link]). Key benefits to adopting an adaptive systems approach are fostering policy and community adjustments to climate change and building interdisciplinary relationships among researchers and community members.

A following project of the CRGC reflected on the organization's own methodologies and practices within the GOM. A cross‐sectional, in‐person study conducted in three CRGC‐partnered communities was used as a case study for this critical reflection. The survey sought to evaluate “the role of social networks, risk perception, preparedness measures, individual resilience, and demographics as predictors of preparedness and resilience” for future disasters in the GOM (Lesen et al., 2019, p. 3). After eight interviews with CHWs who were involved with the study, Lesen et al. (2019) recommended that (1) disaster resilience studies should involve the community as much as possible, as those communities often see the research as valuable; (2) during the development phase, researchers should consult with community partners to avoid cultural conflict and obtain feedback on recruitment and research methodologies; (3) cultural norms and communication methods should be incorporated into researcher‐participant interactions; (4) community partners should be consulted when deciding how to disseminate results to the community; and (5) incorporating critical reflection and considerations of power dynamics in communities that have disaster history can empower community members and researchers by promoting ethically and socially just resilience research (Lesen et al., 2019).

## 7 Studies Listed as Public Health but not Directly Relevant to Human Health

McCoy et al. (2016) authored a tribute to and a review of the scientific legacy of the late Dr. Louis J. Guillette Jr. and his work as a reproductive biologist. Dr. Guillette was an internationally recognized leader in comparative reproductive biology and impacts of endocrine disrupting chemicals to both animals and humans, with prominent works including temperature‐dependent sex determination of reptiles. GoMRI supported some of the recent research conducted by his lab, including work on the American alligator (e.g., see Kohno et al., 2014, Kohno et al., 2015). Dr. Guillette and his colleagues used juvenile alligators as experimental animals to study a variety of factors affecting reproductive health, with some of the work producing information relevant to humans. Dr. Guillette was also a leader in translational research, bridging from animal studies to work on human reproduction and recently including effects of phthalate exposures in pregnant women.

Nine other papers, notably those by Camilli et al. (2012), Deleo et al. (2016), Galligan, Schwacke, Houser, et al. (2018), Galligan, Schwacke, McFee, & Boggs, (2018), Kohno et al. (2014), Kohno et al. (2015), Paruk et al. (2016), Toyota et al. (2016), Washburn et al. (2017), and Zhao et al. (2015), listed in the Public Health category on the GoMRI website were found upon examination not to mention human or public health or have apparent direct relevance to human health. Nonetheless, Deleo et al. and Washburn et al. reported toxic effects of oil or oil‐dispersant mixtures on invertebrate animals; Toyota et al. found that Corexit 9500 was highly toxic to *Daphnia magna* during the offspring growth period at concentrations of 4–64 ppm; and Paruk et al. found that chronic exposure to PAHs was associated with lower body mass in water birds (common loons). While these authors did not consider human health issues, their findings should raise questions about potential toxicity of oil and dispersants to other organisms, including humans. The paper by Camilli et al. examined conflicts between the U.S. legal system and scientific inquiry, and those by Galligan et al. dealt with steroids in marine mammals. Kohno et al. dealt with temperature‐dependent sex determination and its relation to gonad development in reptiles with a view toward increasing understanding of evolution of these phenomena in vertebrates, while Kohno et al. examined how estrogens can affect temperature‐dependent sex determination in the American alligator. Zhao et al. reported on the construction of gold nanoporous materials that can be used in various applications, including in techniques for molecular sensing.

## 8 Human Health‐Related Presentations at GOMOSES Conferences

**Figure 2:**
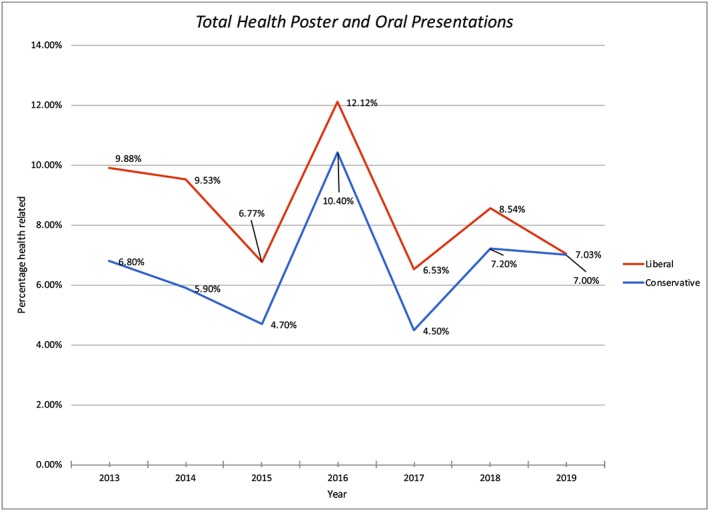
Estimated total human health‐focused oral and poster presentations at annual Gulf of Mexico Oil Spill and Ecosystem Science Conferences, 2013–2019. Red line derived from liberal and blue line from conservative identification methods.

To gain additional insight into public health contributions of the GoMRI, the first (R. E.) and third (P. S.) authors conducted independent reviews of oral and poster presentations at the annual GOMOSES conferences from its inception in 2013 through the February 2019 conference based on information in the published program and data on presentations at each conference derived from the Conference Annual Reports available on the GoMRI website. To identify human health‐focused presentations, R. E. used specific search terms (disaster preparedness, decision making, economy, human health, resilience, community, toxicity, and HABs) while P. S. examined the title of each oral and poster presentations. These two methods resulted in slightly different but overall similar results (Figure 2; Table 1), with human health‐related papers comprising 3.5–13.2% of oral and 0.4–9.2% of poster presentations annually in the more conservative estimates and 7.1–16.7% for oral and 0.4–9.3% for poster presentations annually in the less conservative or liberal counts. Over the 7‐year history of the conference, the number of human health‐related presentations, including both oral and poster, have averaged 6.6–8.6% of total presentations, with a significant increase in the number of presentations focused on human health in 2016, likely as a result of strong efforts by session organizers that year.

**Table 1:** Estimated Numbers of Human Health‐Related Oral and Poster Presentations at Gulf of Mexico Oil Spill and Ecosystem Science Conferences, 2013–2019, Using Liberal and Conservative Counting Criteria

Liberal Counts						
	Oral presentations	Percentage health	Poster presentations	Percentage health	Totals	Percentage totals
Year	(Health/total)	Liberal	(Health/total)	Liberal	(Health/total)	Liberal
2013	34/334	10.18%	17/182	9.34%	51/516	9.88%
2014	31/152	20.39%	22/404	5.45%	53/556	9.53%
2015	18/255	7.06%	12/188	6.38%	30/443	6.77%
2016	47/280	16.79%	16/240	6.67%	63/520	12.12%
2017	33/337	9.79%	6/260	2.31%	39/597	6.53%
2018	27/310	8.71%	11/135	8.15%	38/445	8.54%
2019	34/257	13.20%	1/241	0.40%	35/498	7.03%
Totals	224/1925	11.64%	84/1650	5.09%	309/3575	8.64%
Means	32/275	11.64%	12/235.7	5.09%	44.1/510.7	8.64%
Conservative Counts						
	Oral presentations	Percentage health	Poster presentations	Percentage health	Totals	Percentage totals
Year	(Health/total)	Conservative	(Health/total)	Conservative	(Health/total)	Conservative
2013	22/334	6.59%	13/182	7.10%	35/516	6.80%
2014	18/152	11.84%	15/404	3.70%	33/556	5.90%
2015	9/255	3.53%	12/188	6.40%	21/443	4.70%
2016	32/280	11.43%	22/240	9.20.%	54/520	10.40%
2017	22/337	6.53%	5/260	1.90%	27/597	4.50%
2018	27/310	8.71%	5/135	3.70%	32/445	7.20%
2019	34/257	13.23%	1/241	0.40%	35/498	7.00%
Totals	164/1925	8.52%	73/1650	4.42%	237/3575	6.63%
Means	46.8/275	8.52%	10.4/235.7	4.41%	33.9/510.7	6.64%

## 9 Discussion

The DWH oil spill response and recovery programs, including those directed toward funding of research, have had significant effects on the GOM and its residents. This review focused primarily on published, peer‐reviewed articles produced from research sponsored by GoMRI and identified by GoMRI as having either direct or potential relevance to human health (http://research.gulfresearchinitiative.org). We did not attempt to review the much larger collection of published GoMRI papers dealing with other environmental impacts, the transport and fate of oil, physical oceanography, and additional topics as these have either have been, or are being, reviewed elsewhere (e.g., see Beyer et al., 2016). However, we did enumerate the human health‐related presentations at the GOMOSES conferences.

Taken together, the GoMRI publications constitute a valuable contribution to a scientific understanding of the effect of oil spills on both the environment and humans. While some of the previous oil spills, perhaps most notably the *Exxon Valdez* spill of 1989, resulted in many publications, it is likely that none have surpassed the volume of scientific literature produced following the DWH oil spill.

Some findings from the GoMRI literature dealing with human health deemed significant based on our knowledge of the larger oil spill effects literature and for their potential to influence future oil spill responses are as follows:

1. The large quantities of dispersants (Corexit) used to disperse the oil spilled during the DWH event raised a number of direct and potential human health concerns, including possibilities that compounds comprising the dispersant mixture may be human obesogens and that aerosolization of oil particles mixed with dispersants may impact human health via inhalation.
2. Direct contamination with oil may be related to pulmonary problems in humans, seafood safety issues, potential increase in HABs, and higher populations of pathogenic *Vibrio* bacteria in the environment. While increased contamination of shellfish by *Vibrio* spp. was not observed, tar balls were found to contain larger numbers of pathogenic *Vibrio* spp. and hence may be a potential public health risk, especially from human exposure to highly virulent *V*. *vulnificus*. Buried oil and oil‐dispersant mixtures on beaches were also identified as possible long‐term exposure routes for PAHs and putative obesogens.
3. Those who live in rural communities and are heavily reliant upon natural resource‐based employment and lifestyles were most affected by, and most vulnerable to, disaster‐associated stress and to increased prevalence of depression, anxiety, and alcohol misuse. Those individuals also exhibited greater distrust of the perceived primary responsible party and other institutional players. Trauma history was the most significant predictor of a negative outcome, such as depression, illness anxiety, and alcohol abuse. Communities having a higher level of religious practice may help mitigate negative effects of a disaster, notably for the religious community members, while nonreligious individuals not able to attain such social support reported higher levels of alcohol misuse. Strong community attachment, however, appears to help mitigate impacts, strengthen resilience, and enhance recovery in some circumstances.
4. Several improvements to the U.S. disaster response capacity that can reduce human health impact of a future oil spill or other disasters include (1) a framework for a self‐evaluative and adaptive system, (2) use of messaging methods for risk communication during a disaster event, (3) training and greater use of trusted CHWs, (4) and improving community resilience via an informed focus on stress relief in disaster preparedness, response, and recovery programs.
5. While direct human exposure to contaminants is an important impact of a disaster, such as an oil spill of the magnitude of the DWH, potential mental and behavioral health impacts should also be of primary concern.

The body of work reviewed in this paper has contributed to our understanding of oil spills, their remediation, and their impacts on human health. Future oil spill cleanup efforts and public health responses should take findings from this body of work into consideration so as to improve cleanup methods by reducing use of dispersants or finding less potentially harmful alternatives to reduce human health impacts. They also point to the need for further studies of the risk posed by dispersant exposure to humans and other organisms. The National Academies of Sciences, Engineering and Medicine recently concluded that the use of dispersants is justified in certain circumstances, but they also highlighted the need for additional research (National Academies of Sciences, Engineering and Medicine, 2019).

This assessment of GoMRI public health contributions also points to a major issue namely, that more attention needs to be paid to human health before and after a major oil spill or other disaster. While “impact of oil spills on public health” was identified by the GoMRI as one of its five research themes (http://gulfresearchinitiative.org), the proportion of human health**‐**related papers produced from GoMRI‐supported research to date is relatively modest, comprising only ~3% of the peer‐reviewed publications produced with GoMRI support to date. Although the GoMRI conducted a well‐advertised competition for funding for human health‐oriented projects and provided opportunities for presentation of papers related to human impacts at its annual GOMOSES conference, a stronger focus on research, education, and training related to human health effects of oil spills is essential to deal with future oil spills. Nevertheless, the GoMRI track record is better by nearly a factor of three than the 1% level of human health‐focused studies reported by Murphy et al. (2016) in an extensive review of approximately 10% of all oil spill literature published between 1968 and 2015. Also, at least a portion of the NIH (NIEHS) funding for human health research related to the DWH was supported by a $10 million allocation from the original $45 million block grant referenced earlier. Some of this health effects literature has been reviewed by Laffon et al. (2016), Lichtveld et al. (2016), Croisant et al. (2017), Sandifer et al. (2017), Sandifer and Walker (2018), National Academies of Sciences, Engineering and Medicine (2019), among others. Two large cohort studies related to health effects were also supported by the NIH (the GuLF STUDY, https://gulfstudy.nih; Kwok et al., 2017) and the U.S. Coast Guard Cohort Study (Rusiecki et al., 2018), with papers from these studies continuing to appear.

With the notable exception of the ongoing large cohort studies, most of the human health research efforts external to GoMRI were supported by what appear to be one‐time, multiyear funding, although a number of important studies were supported for multiple years (e.g., see Lichtveld et al., 2016). Yet, none of the funding agencies established ongoing core grant programs specifically targeted to human health effects of oil spills or other disasters. That GoMRI included human health studies as a priority from its beginning and over the entirety of its 10‐year lifespan is praiseworthy. The GoMRI Research Board made a distinct effort to attract and fund research proposals focused directly on human health (R. Colwell GoMRI, personal communication). Yet, it is obvious that human health during and following disasters remains a nascent area of research. Much remains to be done in the future to integrate environmental and human impact studies and especially fund research on human health impacts of oil spills. If similar funding opportunities following disasters occur in the future, additional efforts should be made to engage the public health and biomedical communities early in the development of research plans and funding opportunities. It will also be necessary for the human health research and development community to focus on research priorities and organize effectively. Finally, because human effect studies not related to direct oil exposure are generally not allowed under the implementing regulations of the Oil Spill Act of 1990 (Sandifer & Walker, 2018), it is recommended that Oil Spill Act of 1990 be updated to ensure coverage for direct, indirect, and long‐term effects of oil spills on the mental and physical health of humans.

## Conflict of Interest

The authors declare no conflicts of interest relevant to this study.

## Supporting information



Supporting Information S1
