# Supplementary material for: Oil Spills and Human Health: Contributions of the Gulf of Mexico Research Initiative
Source: Geohealth. 2019 Dec 11;3(12):391–406. doi: 10.1029/2019GH000217 (PMC7038885; doi:10.1029/2019GH000217)
Supplement: Supplementary file 1 — Supporting Information S1 [file GH2-3-391-s001.docx]

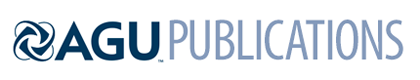


*GeoHealth*

Supporting Information for

**Oil Spills and Human Health: Contributions of the Gulf of Mexico Research Initiative**

Ruth L. Eklund^1^, Landon C. Knapp^2^, Paul A. Sandifer^3^, and Rita C. Colwell^4^

^1^College of Charleston, Masters Programs in Environmental Studies and Public Administration, Charleston, SC, USA.

^2^ College of Charleston, Charleston, SC, USA.

^3^ College of Charleston, Center for Coastal Environmental and Human Health, Charleston, SC, USA.

^4^ Chair, Research Board, Gulf of Mexico Research Initiative and Distinguished Professor, University of Maryland College Park and Johns Hopkins University School of Public Health

**Contents of this file**

Caption for Figure 1

Caption for Figure 2

Caption for Table 1

Supplemental Table 1

Caption for Supplemental Table 1

Caption for Figure 1:

**Figure 1:** Total number of GoMRI-funded publications within each research theme denoted by theme and percent of the total number of publications.

Caption for Figure 2:

Figure 2. Estimated total human health-focused oral and poster presentations at annual Gulf of Mexico Oil Spill and Ecosystem Science Conferences, 2013-2019. Red line derived from liberal and blue line from conservative identification methods.

Caption for Table 1:

Table 1. Estimated numbers of human-health related oral and poster presentations at Gulf of Mexico Oil Spill and Ecosystem Science conferences, 2013-2019, using liberal and conservative counting criteria.

Caption for Supplemental Table 1:

Supplemental Table 1: List of all GoMRI-funded publications included in this review.

Supplemental Table 1

| Afshar-Mohajer, N., Fox, M. A., & Koehler, K. (2019). The human health risk estimation of inhaled oil spill emissions with and without adding dispersant. *Science of The Total Environment*, *654*, 924–932. |
| --- |
| Afshar-Mohajer, N., Li, C., Rule, A. M., Katz, J., & Koehler, K. (2018). A laboratory study of particulate and gaseous emissions from crude oil and crude oil-dispersant contaminated seawater due to breaking waves. *Atmospheric Environment*, *179*, 177–186. |
| Ayer, L., Engel, C., Parker, A., Seelam, R., & Ramchand, R. (In-Press). Behavioral health of Gulf Coast residents six years after the Deepwater Horizon oil spill: the role of trauma history. *Disaster Medicine and Public Health Preparedness*, , 1–7. |
| Baatz, J. E., Newton, D. A., Riemer, E. C., Denlinger, C. E., Jones, E. E., Drake, R. R., et al. (2014). Cryopreservation of Viable Human Lung Tissue for Versatile Post-thaw Analyses and Culture. *In Vivo*, *28*(4), 411–423. |
| Beresford, S., Fillingham, K., & Miller-Way, T. (2018). Recommendations for Science Outreach Program Development: Perspectives from Gulf of Mexico Research Initiative Consortia. *Limnology and Oceanography Bulletin*, *27*(3), 67–75. |
| Bowers, R. R., Temkin, A. M., Guillette, L. J., Baatz, J. E., & Spyropoulos, D. D. (2016). The commonly used nonionic surfactant Span 80 has RXRα transactivation activity, which likely increases the obesogenic potential of oil dispersants and food emulsifiers. *General and Comparative Endocrinology*, *238*, 61–68. |
| Camilli, R., Bowen, A., Reddy, C. M., Seewald, J. S., & Yoerger, D. R. (2012). When Scientific Research and Legal Practice Collide. *Science*, *337*(6102), 1608–1609. |
| Cope, M. R., & Slack, T. (2017). Emplaced social vulnerability to technological disasters: Southeast Louisiana and the BP Deepwater Horizon oil spill. *Popul Environ*, *38*(3), 217–241. |
| Cope, M. R., Slack, T., Blanchard, T. C., & Lee, M. R. (2013). Does time heal all wounds? Community attachment, natural resource employment, and health impacts in the wake of the BP Deepwater Horizon disaster. *Social Science Research*, *42*(3), 872–881. |
| Cope, M. R., Slack, T., Blanchard, T. C., & Lee, M. R. (2016). It's Not Whether You Win or Lose, It's How You Place the Blame: Shifting Perceptions of Recreancy in the Context of the Deepwater Horizon Oil Spill: Shifting Perceptions of Recreancy. *Rural Sociology*, *81*(3), 295–315. |
| DeLeo, D. M., Ruiz-Ramos, D. V., Baums, I. B., & Cordes, E. E. (2016). Response of deep-water corals to oil and chemical dispersant exposure. *Deep Sea Research Part II: Topical Studies in Oceanography*, *129*, 137–147. |
| Dickey, R., & Huettel, M. (2016). Seafood and Beach Safety in the Aftermath of the Deepwater Horizon Oil Spill. *Oceanog*, *29*(3), 196–203. |
| Drakeford, L., Parks, V., Slack, T., Ramchand, R., Finucane, M., & Lee, M. R. (In-Press). Oil Spill Disruption and Problem Drinking: Assessing the Impact of Religious Context among Gulf Coast Residents. *Population Research and Policy Review*, . |
| Finucane, M. L., Blum, M., Ramchand, R., Parker, A. M., Nataraj, S., Clancy, N., et al. (In-Press). Advancing Community Resilience Research and Practice: Moving from “Me” to “We” to “3D”. *Journal of Risk Research*, , 1–10. |
| Galligan, T. M., Schwacke, L. H., Houser, D. S., Wells, R. S., Rowles, T., & Boggs, A. S. P. (2018). Characterization of circulating steroid hormone profiles in the bottlenose dolphin ( Tursiops truncatus ) by liquid chromatography-tandem mass spectrometry (LC-MS/MS). *General and Comparative Endocrinology*, *263*, 80–91. |
| Galligan, T. M., Schwacke, L. H., McFee, W. E., & Boggs, A. S. P. (2018). Evidence for cortisol-cortisone metabolism by marine mammal blubber. *Mar Biol*, *165*(7). |
| Kohno, S., Bernhard, M. C., Katsu, Y., Zhu, J., Bryan, T. A., Doheny, B. M., et al. (2015). Estrogen Receptor 1 (ESR1; ERα), not ESR2 (ERβ), Modulates Estrogen-Induced Sex Reversal in the American Alligator, a Species With Temperature-Dependent Sex Determination. *Endocrinology*, *156*(5), 1887–1899. |
| Kohno, S., Parrott, B. B., Yatsu, R., Miyagawa, S., Moore, B. C., Iguchi, T., et al. (2014). Gonadal Differentiation in Reptiles Exhibiting Environmental Sex Determination. *Sex Dev*, *8*(5), 208–226. |
| Lee, M. R., & Blanchard, T. C. (2012). Community Attachment and Negative Affective States in the Context of the BP Deepwater Horizon Disaster. *American Behavioral Scientist*, *56*(1), 24–47. |
| Lenes, J., Walsh, J., & Darrow, B. (2013). Simulating cell death in the termination of Karenia brevis blooms: implications for predicting aerosol toxicity vectors to humans. *Mar. Ecol. Prog. Ser.*, *493*, 71–81. |
| MacDonald, I. R., Kammen, D. M., & Fan, M. (2014). Science in the aftermath: investigations of the DWH hydrocarbon discharge. *Environ. Res. Lett.*, *9*(12), 125006. |
| McCoy, K. A., Roark, A. M., Boggs, A. S. P., Bowden, J. A., Cruze, L., Edwards, T. M., et al. (2016). Integrative and comparative reproductive biology: From alligators to xenobiotics. *General and Comparative Endocrinology*, *238*, 23–31. |
| Murphy, D., Gemmell, B., Vaccari, L., Li, C., Bacosa, H., Evans, M., et al. (2016). An in-depth survey of the oil spill literature since 1968: Long term trends and changes since Deepwater Horizon. *Marine Pollution Bulletin*, *113*(1-2), 371–379. |
| Nicholls, K., Picou, S. J., & McCord, S. C. (2017). Training Community Health Workers to Enhance Disaster Resilience. *Journal of Public Health Management and Practice*, *23*, S78–S84. |
| Parks, V., Drakeford, L., Cope, M. R., & Slack, T. (2018). Disruption of Routine Behaviors Following the Deepwater Horizon Oil Spill. *Society & Natural Resources*, *31*(3), 277–290. |
| Paruk, J. D., Adams, E. M., Uher-Koch, H., Kovach, K. A., Long IV, D., Perkins, C., et al. (2016). Polycyclic aromatic hydrocarbons in blood related to lower body mass in common loons. *Science of The Total Environment*, *565*, 360–368. |
| Patel, M., Saltzman, L., Ferreira, R., & Lesen, A. (2018). Resilience: Examining the Impacts of the Deepwater Horizon Oil Spill on the Gulf Coast Vietnamese American Community. *Social Sciences*, *7*(10), 203. |
| Sandifer, P., & Walker, A. H. (2018). Enhancing Disaster Resilience by Reducing Stress-Associated Health Impacts. *Frontiers in Public Health*, *6*, 2296–2565. |
| Smith, C. B., Johnson, C. N., & King, G. M. (2011). Assessment of Polyaromatic Hydrocarbon Degradation by Potentially Pathogenic Environmental Vibrio parahaemolyticus Isolates from Coastal Louisiana, USA. *Marine Pollution Bulletin*, *64*(1), 138–143. |
| Tao, Z., Bullard, S., & Arias, C. (2011). High Numbers of Vibrio vulnificus in Tar Balls Collected from Oiled Areas of the North-Central Gulf of Mexico Following the 2010 BP Deepwater Horizon Oil Spill. *EcoHealth*, *8*(4), 507–511. |
| Temkin, A. M., & Spyropoulos, D. D. (2014). Induced pluripotent stem cell technology and aquatic animal species. *Comparative Biochemistry and Physiology Part C: Toxicology & Pharmacology*, *163*(1), 3–13. |
| Temkin, A. M., Bowers, R. R., Magaletta, M. E., Holshouser, S., Maggi, A., Ciana, P., et al. (2016). Effects of Crude Oil/Dispersant Mixture and Dispersant Components on PPARy Activity in Vitro and in Vivo: Identification of Dioctyl Sodium Sulfosuccinate (DOSS; CAS #577-11-7) as a Probable Obesogen. *Ehp*, *124*(1). |
| Toyota, K., McNabb, N. A., Spyropoulos, D. D., Iguchi, T., & Kohno, S. (2016). Toxic effects of chemical dispersant Corexit 9500 on water flea*Daphnia magna*: Toxic effects of corexit 9500 on*DAPHNIA MAGNA*. *J. Appl. Toxicol.*, *37*(2), 201–206. |
| Walsh, J. J., Lenes, J. M., Darrow, B., Parks, A., & Weisberg, R. H. (2016). Impacts of combined overfishing and oil spills on the plankton trophodynamics of the West Florida shelf over the last half century of 1965-2011: A two-dimensional simulation analysis of biotic state transitions, from a zooplankton- to a bacterioplankton-modulated ecosystem. *Continental Shelf Research*, *116*, 54–73. |
| Walsh, J. J., Lenes, J. M., Weisberg, R. H., Zheng, L., Hu, C., Fanning, K. A., et al. (2017). More surprises in the global greenhouse: Human health impacts from recent toxic marine aerosol formations, due to centennial alterations of world-wide coastal food webs. *Marine Pollution Bulletin*, *116*(1-2), 9–40. |
| Washburn, T. W., Reuscher, M. G., Montagna, P. A., Cooksey, C., & Hyland, J. L. (2017). Macrobenthic community structure in the deep Gulf of Mexico one year after the Deepwater Horizon blowout. *Deep Sea Research Part I: Oceanographic Research Papers*, *127*, 21–30. |
| Weisberg, R. H., Zheng, L., Liu, Y., Corcoran, A. A., Lembke, C., Hu, C., et al. (2016). Karenia brevis blooms on the West Florida Shelf: A comparative study of the robust 2012 bloom and the nearly null 2013 event. *Continental Shelf Research*, *120*, 106–121. |
| Xia, K., Hagood, G., Childers, C., Atkins, J., Rogers, B., Ware, L., et al. (2012). Polycyclic Aromatic Hydrocarbons (PAHs) in Mississippi Seafood from Areas Affected by the Deepwater Horizon Oil Spill. *Environ. Sci. Technol.*, *46*(10), 5310–5318. |
| Zhao, F., Zeng, J., Santos, G. M., & Shih, W. - C. (2015). In situ patterning of hierarchical nanoporous gold structures by in-plane dealloying. *Materials Science and Engineering: B*, *194*, 34–40. |
